# Usage of Traditional and Complementary Medicine among Dengue Fever Patients in the Northeast Region of Peninsular Malaysia

**DOI:** 10.21315/mjms2019.26.3.7

**Published:** 2019-06-28

**Authors:** Ida Seriwati Ismail, Suhaily Mohd Hairon, Najib Majdi Yaacob, Alwi Muhd Besari, Sarimah Abdullah

**Affiliations:** 1 Unit of Biostatistics & Research Methodology, School of Medical Sciences, Universiti Sains Malaysia, Kubang Kerian, Kelantan, Malaysia; 2 Department of Community Medicine, School of Medical Sciences, Universiti Sains Malaysia, Kubang Kerian, Kelantan, Malaysia; 3 Department of Medicine, School of Medical Sciences, Universiti Sains Malaysia, Kubang Kerian, Kelantan, Malaysia

**Keywords:** dengue fever, traditional and complementary medicine, crab soup, papaya leaf extract, isotonic drinks

## Abstract

**Background:**

The recent epidemic of dengue fever (DF) in Malaysia was alarming. The treatment of DF remains supportive as there is no anti-viral agent or vaccine available as yet. Traditional and complementary medicine (T&CM) provides an alternative option for the treatment of DF but there is limited evidence with regard to its usage. The aim of this study was to determine the prevalence, types and predictor factors of T&CM usage among DF patients in the northeast region of Peninsular Malaysia.

**Methodology:**

This was a cross-sectional study of DF patients in the northeast region of Peninsular Malaysia who had been admitted to a tertiary centre from January 2014 until December 2015. Serologically-confirmed DF patients aged 18 years and above were randomly selected. Phone interviews were conducted to obtain information regarding the use of T&CM during hospitalisation. Notes were made regarding the prevalence and type of T&CM used. Binary logistic regressions were used to identify the predictor factors of T&CM usage.

**Results:**

A total of 241 DF patients with a mean age of 36.62 (SD = 14.62) years were included. The estimated prevalence of T&CM usage was 84.6% (95%CI: 80.1%, 89.2%). The most common T&CM used were crab soup (85.3%), papaya leaf extract (64.2%) and isotonic drinks (61.8%). The significant predictors for T&CM usage were age [adjusted odds ratio (AOR) 0.97; 95%CI: 0.94, 0.99], tertiary education (AOR 3.86; 95%CI: 1.21, 12.32) and unemployment (AOR 2.55; 95%CI: 1.02, 6.42).

**Conclusion:**

The prevalence of T&CM usage in our population is high. Age, tertiary education and unemployment influence the use of T&CM.

## Introduction

Dengue fever (DF) is one of the most important mosquito-borne viral diseases, and it is transmitted by either the *Aedes (Stegomyia) aegypti* or *Aedes (Stegomyia) albopictus* (1, 2). There are four antigenically distinct but related dengue viruses (DENV 1–4) which cause infections that are symptomatic or asymptomatic with seroconversion (3). The symptomatic infections can lead to systemic diseases with both severe and non-severe manifestations (4, 5).

Although the Flavivirus genus represents more than 70 viruses, dengue has the highest impact in terms of global disease incidence (6, 7). It is estimated that there are 390 million dengue infections per year, with 294 million asymptomatic, undetected infections having occurred worldwide in 2010 (8). In Malaysia, dengue is perceived as a highly contagious health threat with an escalating trend of infection, where 14% of dengue cases and a death toll of 8% per annum occurred during the period 2000 to 2010 (2). Furthermore, the dengue incidence rate was amplified by nearly 250% in 2014 and continued to rise the following year (9).

The global burden of dengue is alarming and poses an increasing challenge to public health officials and policymakers (8). Current efforts to control the transmission of dengue using chemical, biological and environmental management methods are mainly focused on the vector and its breeding sites (10). However, these approaches have failed to reduce the incidence, epidemics and widespread geographical transmission of dengue fever (3, 8, 9). To date, no anti-viral agent has been developed to treat dengue infections, and thus, the treatment remains supportive, and includes the administration of paracetamol and isotonic intravenous fluids, and close monitoring of blood glucose and platelet levels (4, 5).

The lack of anti-viral drugs and vaccines against dengue infections has motivated the public to rely on traditional practices and home remedies (11, 12). The World Health Organization (WHO) defines traditional medicine as ‘the sum total of knowledge, skills and practices based on the theories, beliefs and experiences indigenous to different cultures, whether explicable or not, used in the maintenance of health, as well as in the prevention, diagnosis, improvement or treatment of physical and mental illness’. Complementary medicine is defined as ‘a broad set of health care practices that are not part of that country’s own tradition or conventional medicine’ (13).

In some countries, the terms complementary medicine, alternative medicine, and non-conventional medicine are used interchangeably. Meanwhile, in Malaysia, the term traditional and complementary medicine (T&CM) is used to denote a practice of medicine that is other than the medical or dental practices utilised by registered medical or dental practitioners (14). T&CM can be classified into four types, namely, whole medical systems, Islamic medical practices, homeopathy and complementary medicine. Complementary medicine can be further divided into biological-based practices, manipulative-based practices, energy medicine, and mind, body and soul therapy.

T&CM is accessible, affordable and widely used in developing countries, including Malaysia. According to Siti et al. (15), the prevalence of T&CM usage in Malaysia is between 50%– 80%, as estimated by the WHO. It has become an alternative approach to the health care system, where the society, being concerned about the adverse effects of chemical drugs, is questioning the approaches and assumptions of allopathic medicine (16, 17). T&CM is mainly self-prescribed, and is used for the maintenance of health, disease prevention and the treatment of health problems, especially chronic illnesses (15, 18, 19). As for dengue, Kadir et al. (20) reported that 31 species of plants have been found to have the potential to treat dengue infections, although only a few with their isolated compounds have been investigated in vivo and in vitro such as *Carica papaya* leaf, *Euphorbia hirta* (or “tawa-tawa”) and *Andrographis paniculate* (or “hempedu bumi”). While previous studies have reported on T&CM usage in the Western Region of Peninsular Malaysia, this research explored the prevalence and the various types of T&CM available in the northeast region of Peninsular Malaysia, which is mainly populated by the Malay ethnic group. This research also explored the factors associated with T&CM usage.

## Methodology

### Study Setting

This was a cross-sectional study of dengue fever patients admitted to Hospital Universiti Sains Malaysia (HUSM), Kelantan, Malaysia, which was conducted from September 2015 to February 2016. The northeast region of Peninsular Malaysia consists of three states, Kelantan, Terengganu and Pahang. This hospital receives referrals from the entire state of Kelantan and the northern part of Terengganu.

### Inclusion Criteria

All DF patients aged 18 years and above who were admitted to HUSM from January 2014 to December 2015 were eligible for the study. Their infection with DF was confirmed by the IgG, IgM and/or NS1 antigens. Patients with missing contact numbers were excluded from this study.

### Sample Size

The sample size was calculated based on the reported prevalence of T&CM usage among DF patients in Malaysian hospitals by Hasan et al. (18) using a standard formula for the estimation of the proportion of the population. The sample size was calculated to be 190, with a precision of 5%, and a significance level of 5%. Taking into consideration a non-response rate of 40% (due to the failure of the respondents to answer the phone call or their unwillingness to participate in this study), the required sample size was 316.

### Data Collection Method

The list of DF patients admitted to HUSM from January 2014 to December 2015 was obtained from the Medical Records Department of the hospital. Out of the 1351 cases admitted for DF, 1172 of them fulfilled the inclusion criteria. From the list of proposed subjects for the study, 316 patients were randomly selected using a simple random sampling method.

The selected participants were contacted via recorded phone interviews to obtain information regarding the use of T&CM and their socio-demographic characteristics. The patients were asked about any T&CM usage in terms of the types and preparation of T&CM. Each interview session took about 10–15 min. To differentiate between the usual food taken daily and food taken for the purpose of T&CM use, the study participants were asked, “Did you use the food (soup, juice or fruit) for medicinal purposes or to improve your health during your admission for DF?” The participants were informed regarding the research prior to their participation, and their verbal consent was obtained before the phone interviews were conducted. All the phone conversations were recorded and the participants were also assured that any information obtained would remain private and confidential. Their participation in this was on a voluntary basis.

### Statistical Analysis

The data entry and analysis were done by using the SPSS version 22.0. The data were presented as the mean and standard deviation (SD) for the numerical variables; and as the frequency and percentage (%) for the categorical variables. The prevalence of T&CM usage among DF patients was calculated with a confidence interval (CI) of 95%. A stepwise binary logistic regression analysis was used to evaluate the factors associated with T&CM usage among DF patients. The outcome variable was binary, namely, ‘did not use T&CM’, and ‘used T&CM’. The fitness of the model was tested using the Hosmer-Lemeshow goodness of fit test, a classification table and a receiver operating characteristic curve (ROC). The influential outliers were identified using Cook’s influential statistics. The significance level was set at 0.05.

### Operational Definition

In this study, T&CM users were defined as patients who consumed T&CM at least once daily for three consecutive days, and concurrently received the standard management as per the National Clinical Practice Guidelines for the Management of Dengue (21).

## Results

A total of 316 patients were randomly selected from a list of DF patients admitted to HUSM from January 2014 to December 2015. Out of the 316 patients, only 241 responded to the phone call and agreed to participate in this study (response rate = 76.3%). Sixty-four patients did not answer the call, while 29 had given the wrong telephone number.

The mean age of the participants was 36.62 (SD = 14.62) years (ranging from 18 to 79 years). The majority of the participants were male (57.7%), Malay (94.2%), with tertiary education (50.6%) and employed (62.7%). The participants mostly had DF with warning signs (57.7%). The mean length of stay was 4.95 (SD = 1.34) days and the patients who were admitted had 4.71 (SD = 1.58) days of fever, as shown in Table 1. The percentage of DF patients admitted to USM who used T&CM was 84.6%, the majority of whom took more than one type of T&CM (70.5%) (Table 2). Table 3 shows the types of T&CM used by the participants. The total percentage exceeded 100% as each user may have taken more than one type of T&CM. Biological-based practices were the most commonly used typed of T&CM, where the majority of the respondents took crab soup (85.3%) and papaya leaf extract (64.2%). Among the users, 61.8% of them took isotonic drinks and fruit juices such as kiwi (19.6%), coconut (5.9%), guava (3.9%), orange (2.9%) and date (2.5%) juice. They also consumed fruits, namely watermelon (5.4%), grape (5.4%) and apple (2.0%). Fruits were also regarded as a type of T&CM since the users only took them when they contracted DF in the belief that the fruits could resolve the fever.

In the univariate analysis (Table 4), there was no significant association between T&CM usage with gender, race, type of DF and days of fever on admission. A multiple logistic regression was used to identify the predictor factors for T&CM usage. All the significant variables with a *P*-value of less than 0.25 from the univariate analysis, namely, age, education level and employment status, were selected for the multiple logistic regression. From the multiple logistic regression analysis, the significant adjusted predictors for T&CM usage were age (AOR 0.97; 95%CI: 0.94, 0.99; *P* = 0.014), tertiary education (AOR 3.86; 95%CI: 1.21, 12.32; *P* = 0.023) and unemployment (AOR 2.55; 95%CI: 1.02, 6.42; *P* = 0.046) (Table 5).

## Discussion

In the current study, the prevalence of T&CM usage among DF patients in the east coast of Peninsular Malaysia was 84.6%. The high percentage of T&CM usage was almost similar to that obtained by Ching et al. (22), who reported a T&CM usage of 85.3% in the central region of Peninsular Malaysia. The sample for the current research was comparable, whereby almost 80% of the respondents were Malays, aged 18 years and above, and were employed. Thus, the findings were almost similar.

Siti et al. (15), on the other hand, investigated the use of T&CM by the Malaysian public. The sample was taken from the whole of Malaysia, and it was stratified according to urban and rural areas. The prevalence of T&CM usage among the Malaysian population was reported to be 69.4%, a lower prevalence compared to the current research. The T&CM usage was for maintaining the health status and overcoming health issues.

In Singapore, the prevalence of T&CM among the general population was also lower compared to the current study (76%). The usage of T&CM among the Chinese was 84.0%, while among the Malays and Indians, it was 69.0%. Since the Chinese are the predominant ethic group in Singapore, traditional Chinese medicine was the most frequently used type of T&CM (23). The prevalence of T&CM usage was also lower in Japan (76.0%) and South Korea (29%–83%) (24, 25). The prevalence of T&CM usage was lower in these studies because the main purpose of T&CM usage among the general population was more towards the maintenance of health rather than the treatment of disease.

With regard to the types of T&CM used, the majority of the users consumed more than one type of T&CM, and hence, the total percentage of the type of T&CM used exceeded 100%. Seven different varieties of T&CM were used the most, but the majority of the respondents took two types of T&CM (*n* = 60, 24.9%).

Biological-based practices were the type of T&CM that was most used by the DF patients in this study. Similarly, Siti et al. (15) also reported that the majority (88.9%) of the T&CM users preferred biological-based therapies for their health problems, while Ching et al. (22) stated that all their T&CM users (100%) used biological-based therapies.

In the current study, crab soup (84.0%) was the biological-based therapy that was most commonly-used by the DF patients, followed by *Carica papaya* leaf juice (64.2%), and isotonic drinks (61.8%). On the other hand, Ching et al. (22) identified isotonic drinks as having the highest prevalence (85.8%) among their DF patients compared to crab soup (46.7%) and *Carica papaya* leaf juice (22.2%). This trend of taking soups, juices and isotonic drinks as complementary treatments for DF corresponded to the dengue management guidelines by the World Health Organization (4), which encourage the replacement of loss of fluids due to fever and vomiting with adequate oral fluids. The preference for fluids such as soups, fruit juices, or coconut water is optional to cultural acceptance. However, commercial carbonated drinks that exceed the isotonic level (5% sugar) should be avoided as these can exacerbate hyperglycaemia due to physiological stress from dengue and diabetes mellitus (26, 27). Although there is limited evidence as to the efficiency of crab soup, *Carica papaya* leaf juice, and isotonic drinks, the difference in the T&CM preference was possibly influenced by the perceived helpfulness and positive feedback from other T&CM users among DF patients, and the knowledge that there is no specific cure for dengue (11, 22).

Age was found to be a significant predictor of T&CM usage among DF patients in the East Coast of Peninsular Malaysia, with a higher prevalence of T&CM usage among younger patients, with adjustments for education and employment. Whether T&CM is used among younger people is still a debate. One study reported a greater prevalence of T&CM usage among younger people (28), while other studies reported an inverse U-shape relationship between age and the use of T&CM, indicating that there is less usage among younger and older people (29). Hess et al. (28) suggested that the higher incidence of comorbidities among the older age groups hinders the use of T&CM among these people. Meanwhile, an open-minded attitude towards new treatments and a high level of curiosity encourage the use of T&CM among younger people. One of the possible explanations given in this study is that younger patients tend to seek more information regarding T&CM and therefore, they are encouraged to use T&CM to treat their illness. Among the older age groups, comorbidities such as diabetes make them more cautious in using T&CM.

In the current study, unemployment was also found to be a significant predictor of T&CM usage, with adjustments made for age and education level. It was found those who were unemployed were 2.5 times more likely to use T&CM compared to those who were employed. An incentive for the use of T&CM was the cost of seeking treatment (30). As unemployment reflects a lower income, it was not surprising that this group of people tended to seek cheaper treatments such as T&CM. Most of the T&CM users, especially those in the rural and remote areas of Malaysia, were known to prefer relatively inexpensive methods (31). Unemployment was also reported in several previous studies to be a predictor of T&CM usage (32, 33).

Those who had received tertiary education were more likely to use T&CM compared to the other respondents in the current study. This might have been because they were more highly educated and were aware of the complications of dengue, and this made them look for supplements to complement the treatment they received in the hospital. This finding was consistent with that of a few other studies, which demonstrated that individuals with a higher level of education possessed better knowledge of certain diseases, thereby leading them be more aware of their own health (22).

### Strength and Limitations

This study required that the patients be contacted through telephone interviews to obtain certain information, where the number of missing contact details, wrong contact numbers and no answers from the participants made the study more challenging. Another limitation was the potential for recall bias as the participants tended to exaggerate or underrate certain interesting experiences. However, it was not possible to make a comparison between the self-reported data and medical records as the intake of T&CM was not reported to the medical staff or documented in the medical records. Nevertheless, a retrospective study on physical activity and myocardial infarction found that there was no recall bias in the dichotomous-level questions, but it was more likely to occur when the participants were asked more detailed questions that required them to distinguish and specify certain information. This study only asked about T&CM usage and its types (34). The types of T&CM used by the different races are of interest as Malaysia is a multi-ethnic country. However, since the study population was predominated by Malays, this study was unable to describe the use of T&CM according to race as the number of non-Malays who participated in this study was small.

## Conclusion

T&CM usage is high among DF patients. Among the most common T&CM used are crab soup, papaya leaf extract, and isotonic drinks. Tertiary education level, unemployment and younger age were found to be predictor factors of T&CM usage. The potency and efficacy of each type of T&CM should be investigated further to explore the potential use of T&CM for the treatment of DF.

## Figures and Tables

**Table 1:** Descriptive statistics of all study participants (*n* = 241)

Variables	Mean (SD)	*n* (%)
Age	36.62 (14.62)	
Gender		
Male		139 (57.7)
Female		102 (42.3)
Race		
Malay		227 (94.2)
Chinese		7 (2.9)
Indian		4 (1.7)
Others		3 (1.2)
Education		
No formal education		4 (1.7)
Primary		15 (6.2)
Secondary		100 (41.5)
Tertiary		122 (50.6)
Employment		
Employed		151 (62.7)
Unemployed		90 (37.3)
Type of DF		
DF without warning sign		80 (33.2)
DF with warning sign		139 (57.7)
Severe dengue		22 (9.1)
Days of fever on admission (days)	5.00 (2.00)*	
Length of stay (days)	5.00 (2.00)*	

* median (IQR)

**Table 2:** Usage of T&CM among DF patients (*n* = 241)

Variables	*n* (%)
T&CM Usage	
No	37 (15.4)
Yes	204 (84.6)
Number of T&CM used	
0	37 (15.4)
1	34 (14.1)
2	60 (24.9)
3	57 (23.7)
4	40 (16.6)
5	7 (2.9)
6	2 (0.8)
7	4 (1.7)
Days of fever starting T&CM use (*n* = 204)	
Day 1	141 (58.5)
Day 2	27 (11.2)
Day 3	20 (8.3)
Day 4	14 (5.8)
Day 5	2 (0.8)

**Table 3:** Types of T&CM use among DF patients (*n* = 204)

Types of T&CM use	*n* (%)
Traditional Medicine	
Traditional Chinese Medicine	
Chinese medication	1 (0.5)
Chinese herbal drink	1 (0.5)
Traditional Islamic Medical Practice	
*Nigella sativa* (Habbatussauda)	1 (0.5)
Elixir drink (Air penawar)	1 (0.5)
Zamzam water	1 (0.5)
Complementary Medicine	
Biological based therapies	
Animal based	
Crab soup	174 (85.3)
Eel soup	8 (3.9)
Plant based	
Extract	
Papaya leaf extract	131 (64.2)
Juice	
Kiwi juice	40 (19.6)
Coconut juice	12 (5.9)
Guava juice	8 (3.9)
Orange juice	6 (2.9)
Dates juice	5 (2.5)
*Aloe vera* juice	3 (1.5)
Beetroot juice	3 (1.5)
*Cantella asiatica* juice	3 (1.5)
Soybean milk	1 (0.5)
Fruits	
Watermelon	11 (5.4)
Grape	5 (2.5)
Apple	4 (2.0)
Pomegranate	4 (2.0)
Banana	1 (0.5)
Dietary supplement and others	
Isotonic drink	126 (61.8)
Vitamin supplement	4 (2.0)
Milk	3 (1.5)

* Total is more than 100% because one patient can take more than one type of T&CM

**Table 4:** Simple logistic regression for predictors of T&CM use among DF patients (n = 204)

Variables	Group, *n* (%)		Crude b	Crude OR (95%CI)	Wald stat (df = 1)	*P*-value
	T&CM non-user	T&CM user				
Age (years) a	42.62 (16.34)	35.53 (14.06)	−0.03	0.97 (0.95, 0.99)	7.07	0.008
Gender						
Male	21 (56.8)	118 (57.8)	0	1		
Female	16 (43.2)	86 (42.2)	−0.04	0.96 (0.47, 1.94)	0.02	0.902
Race						
Malay	34 (91.8)	193 (94.6)	0			
Non-Malay	3 (8.2)	11 (5.4)	−0.44	0.65 (0.17, 2.44)	0.42	0.519
Education						
Primary and lower	7 (18.9)	12 (5.9)	0	1		
Secondary	18 (48.6)	82 (40.2)	0.98	2.66 (0.92, 7.69)	3.25	0.071
Tertiary	12 (32.5)	110 (53.9)	1.68	5.35 (1.77, 16.17)	8.82	0.003
Employment						
Employed	27 (73.0)	124 (60.8)	0	1		
Unemployed	10 (27.0)	80 (39.2)	0.56	1.74 (0.80, 3.79)	1.95	0.162
Type of DF						
Without warning sign	9 (24.3)	71 (34.8)	0	1		
With warning sign	23 (62.2)	116 (56.9)	−0.39	0.67 (0.23, 2.01)	0.50	0.479
Severe dengue	5 (13.5)	17 (8.3)	0.45	1.56 (0.69, 3.57)	1.13	0.288
Days of fever on admission b	5.00 (2.00)	5.00 (2.00)	−0.01	0.99 (0.79, 1.23)	0.01	0.919

a Mean = SD,

b Median = Interquartile Range (IQR)

**Table 5:** Multiple logistic regression for predictors of T&CM use among DF patients (*n* = 204)

Variables	Group, *n* (%)		Adj. b	Adj. OR (95%CI)	Wald stat (df = 1)	*P*-value
	T&CM non-user	T&CM user				
Age (years) a	42.62 (16.34)	35.53 (14.06)	−0.04	0.97 (0.94, 0.99)	6.07	0.014
Education						
Primary and lower	7 (18.9)	12 (5.9)	0	1		
Secondary	18 (48.6)	82 (40.2)	0.82	2.28 (0.74, 6.93)	2.03	0.154
Tertiary	12 (32.5)	110 (53.9)	1.35	3.86 (1.21, 12.32)	5.21	0.023
Employment						
Employed	27 (73.0)	124 (60.8)	0	1		
Unemployed	10 (27.0)	80 (39.2)	0.94	2.55 (1.02, 6.42)	3.98	0.046

a Mean = SD

Backward LR method used for variable selection Constant = 1.81

No interaction and multicollinearity between age, education and employment

Hosmer and Lemeshow test, *P*-value = 0.113

Classification table overall percentage correct = 84.

Area under ROC cure = 69.2%, 95%CI: 58.9%, 79.6%, *P* < 0.001

Cook’s influential statistics indicate no influential outlier
